# The experience of being a participant in one’s own care at discharge and at home, following a severe acute exacerbation in chronic obstructive pulmonary disease: a longitudinal study

**DOI:** 10.1080/17482631.2017.1371994

**Published:** 2017-09-06

**Authors:** Ingrid Charlotte Andersen, Thora Grothe Thomsen, Poul Bruun, Uffe Bødtger, Lise Hounsgaard

**Affiliations:** ^a^ Odense Patient data Explorative Network, Odense University Hospital/Department of Clinical Research, University of Southern Denmark, Odense, Denmark; ^b^ Department of Medicine, Slagelse Hospital, Slagelse, Denmark; ^c^ Zealand University Hospital, Roskilde and Koege, Denmark and Institute of Regional Health Research, University of Southern Denmark, OdenseDenmark; ^d^ Health Sciences Research Center, University College Lillebaelt, Denmark; ^e^ Department of Respiratory Medicine, Næstved Hospital, Næstved, Denmark and Institute of Regional Health Research, University of Southern Denmark, Odense, Denmark

**Keywords:** Chronic obstructive pulmonary disease, hospital discharge, follow-up, patient participation, self-management, patients’ lived experiences, phenomenological-hermeneutic research

## Abstract

**Purpose:** In healthcare related to hospital discharge and follow-up, it is acknowledged that patient participation can strengthen self-management in patients with chronic obstructive pulmonary disease. However, the meaning of participation in care following a severe acute exacerbation is less described. Therefore, the aim of this part of a larger study was to explore patients’ experiences of participating in their care around discharge and in their subsequent day-to-day care at home. **Method:** The study was designed as a qualitative, longitudinal study. Data were collected by repeated participant observations and in-depth interviews with 15 patients within a period of 18 months post-discharge. A phenomenological-hermeneutic approach was used to interpret the data. **Results:** Before discharge, the patients struggled to regain a sense of control in their efforts to build up strength, and acquire sufficient clarity and confidence to face self-management at home. At home, the patients strived to comply with advice and encouragement in a struggle to stay motivated and confident, and to ask for help. **Conclusions:** With more knowledge about patients’ participation in care, healthcare professionals can encounter patients in ways that are sensitive to their specific care and support needs and, thereby, contribute to the promotion of patients’ health and well‐being.

## Introduction

This article addresses what it means to patients with chronic obstructive pulmonary disease (COPD) to participate in their own care in the transitional period that includes discharge, follow-up visits and subsequent everyday life at home following a hospitalization for a severe acute exacerbation in chronic obstructive pulmonary disease (AECOPD). Chronic obstructive pulmonary disease is an often progressive life-threatening lung disease that represents a major cause of morbidity and mortality worldwide; it imposes a considerable burden on society all over the world and its incidence and prevalence is likely to increase in the coming years because of higher smoking prevalence and ageing populations in many countries (World Health Organization, 2017). Knowledge about the physical and psychosocial consequences of living with COPD is growing, and globally there is an ongoing focus on the optimization of its treatment and management (Global Initiative for Chronic Obstructive Lung Disease (GOLD), 2017).

Integral to this development are the strategies employed by healthcare professionals to engage, support and empower patients to have a central role in improving health outcomes, quality of life, patient security and reduced costs in health services (Castro, Van Regenmortel, Vanhaecht, Sermeus, & Van Hecke, 2016; Coulter, 2011). Patient participation is one of the many concepts used to describe this trend. In this study, the term is understood as proposed by Castro et al. (2016): “a patient’s right and opportunities to influence and engage in the decision making about his own care through a dialogue attuned to his preferences, potential and a combination of his experiential and the professional’s expert knowledge” (p. 1929). For patients, this prepares the ground for active participation in self-management of their condition (Coulter, 2011). However, difficulties related to attitudes, the relationship between patients and healthcare professionals and busy workloads are found by healthcare professionals to be barriers to such patient participation (Angel & Frederiksen, 2015; Tobiano, Marshall, Bucknall, & Chaboyer, 2015). The challenge, therefore, is to understand how to provide meaningful care to successfully work in partnership with patients (Maslowski, 2012). Accordingly, further insight that includes patients’ experiences might help us more clearly understand the complex nature of patients’ participation in their own care.

A distinctive feature of COPD is that symptoms may vary frequently because of exacerbations and disease progression. Besides, COPD often coexists with comorbidities, and this could have a significant impact on prognosis (GOLD, 2017). As the illness progresses, recurring periods of AECOPD can challenge usual day-to-day care and the need for hospitalization can increase (GOLD, 2017). While an admission for an AECOPD can be frightening and demanding, recovery after discharge can also be challenging—sometimes to the extent that it leads to readmission (Giacomini, DeJean, Simeonov, & Smith, 2012; Kvangarsnes, Torheim, Hole, & Ohlund, 2013b). Readmissions are considered a burden by patients and the health economic consequences are considerable (Steer, Gibson, & Bourke, 2010). Because of these challenges, the reduction of admissions and readmissions has become an international focus area (Steer et al., 2010; Steiner, 2015). Besides the ongoing optimization of medical treatments, optimized discharge planning is seen as a crucial component in the work to reduce hospitalization and readmissions (Abad-Corpa et al., 2013). After discharge, it is acknowledged that follow-up and rehabilitation interventions are important to support further self-management and palliative care needs, if required (Bourbeau, 2008; GOLD, 2017; Steiner, 2015). In Denmark, this is put into practice through formalized cooperation between general practice, hospitals and municipalities in accordance with the “Chronic Care Model” guidelines for an integrated health system (Lange, 2012; Wagner, 1998). The model advocates an active role for patients, who are encouraged to become more self-managing and more actively involved in decisions about their care (Coulter et al., 2015). Taking the changing and unpredictable situation after an AECOPD into consideration, a further exploration of patients’ experiences related to the clinical pathway towards everyday life with COPD seems to be warranted.

According to a systematic literature search conducted in February 2017 in CINAHL, Cochrane Library, Medline and PsycINFO, knowledge within this area is growing. With regard to COPD patients’ experiences of their participation in clinical care settings in hospital, the current research points to a range of challenges related to specific settings and situations. In research conducted in an intensive care unit, low levels of power and feelings of being dependent on and ignored by healthcare professionals are found to make patient participation difficult (Kvangarsnes, Torheim, Hole, & Ohlund, 2013a; Torheim & Kvangarsnes, 2014). Other studies show similar challenging perceptions during treatment using non-invasive ventilation (Sørensen, Frederiksen, Grøfte, & Lomborg, 2013) and during assisted personal body care (Jensen, Vedelø, & Lomborg, 2013; Lomborg & Kirkevold, 2008). As shown in a study conducted on a medical ward, lack of attention after the initial symptoms were stabilized, insufficient communication of care plans and lack of coordination between the primary and secondary sectors can make recovery problematic for patients with severe COPD or lung cancer (Bailey, Hewison, Karasouli, Staniszewska, & Munday, 2016). In addition, patients suffering from respiratory disease can be challenged when their need to share their stories about issues related to their coping with illness or existential aspects of their everyday life is not adequately recognized by healthcare professionals during outpatient consultations (Jensen, Brinkjaer, Larsen, & Konradsen, 2015). Such experiences seem to be critical during the transitional period when COPD patients are struggling to stay engaged in their daily self-management, and implementation of effective self-management interventions remains a challenge (Bourbeau et al., 2013; Chen, Chen, Lee, Cho, & Weng, 2008; Chen, Liu, ShyuYea-Ing, & Yeh, 2016; Disler et al., 2016; Disler, Gallagher, & Davidson, 2012; Harrison, Janaudis-Ferreira, Brooks, Desveaux, & Goldstein, 2015; Jolly et al., 2016; Jonkman et al., 2016; Jonsdottir, 2013; Korpershoek et al., 2016; Zwerink et al., 2014). These studies report the experiences of COPD patients related to time-limited settings after an AECOPD. Therefore, taking the intentions of more integrative care into account, this study intends to inform us of the breadth of that experience over time.

## Aim

To explore COPD patients’ experiences of participating in their care in the transitional period around discharge from hospital and in their own subsequent day-to-day care at home following a severe AECOPD.

## Methods

### Design

A phenomenological-hermeneutic approach inspired by the French philosopher Paul Ricoeur’s work around narrative and interpretation guided the study (Dreyer & Pedersen, 2009; Ricoeur, 1976). The design was longitudinal, and ethnographically-inspired fieldwork was used to explore the COPD illness and treatment trajectory during and after hospitalization for a severe AECOPD (Hounsgaard, Petersen, & Pedersen, 2007; Murray et al., 2009; Saldaña, 2003; Spradley, 1980). The study was conducted in a department of respiratory medicine at a regional acute hospital in rural Denmark and in the participants’ own homes. In the department in which this study was conducted, the clinical pathway is organized so that, after being stabilized in an acute care or intensive care setting, patients are transferred to a respiratory medical ward for further treatment, stabilization and follow up on discharge. This study is part of a larger, explorative study that involves both a patient and family perspective. Results concerning the meaning of family participation in care will be published separately. This article focuses solely on exploring the significance of patients’ experiences in going through the phases in the clinical pathway that include discharge, hospital follow-up visits and subsequent day-to-day care at home.

### The clinical pathway

The treatment and management of COPD in Denmark follows recommendations made in national and regional disease management programmes that describe optimal care for COPD (Lange, 2012). Patients with an AECOPD of a severity requiring hospital admission go through a clinical pathway adjusted to individual needs for pharmacological and non-pharmacological therapies (GOLD, 2017). In Denmark in 2014, the mean length of stay in hospital after an AECOPD was 5.4 days and a 30-day readmission rate 19% (Danish Health Authority, 2015). Discharge from hospital is a multi-interventional process, in which elements such as optimization of medication, education, supervision of inhaler techniques and coordination of different kinds of activities, including follow-up, tele-monitoring and rehabilitation activities are recommended (GOLD, 2017).

### Participants

Participants were included based on inclusion criteria: doctor’s diagnosis of COPD, hospitalized due to an AECOPD and Danish-speaking. Medically unstable or palliative care patients were excluded. The inclusion of participants took place over a period of six months, in cooperation with the local staff, after stabilization of the acute phase of AECOPD. Participants who fulfilled the inclusion criteria were consecutively recruited. As the study proceeded, the principles of purposeful sampling were employed to achieve the best possible diversity among participants (Polit & Beck, 2006). In total, 15 out of 16 invited patients agreed to participate. For further patient characteristics, see Table 1.

**Table 1.** Baseline demographics of the participants.

*N* = 15	Gender	Age	Classification of COPD *	Impact of COPD CAT score (COPD Assessment Test)**	Comorbidity Charlson Index of comorbidity Score***	History of admissions ****	History of readmissions *****	Smoking status	Profession	Cohabitation status
P1	Female	73	2, D	16	1	No	No	Ex	Retired	Married
P2	Female	56	3, D	17	1	No	No	Ex	Unemployed	Single
P3	Female	55	2, D	11	1	No	No	Ex	Retired	Single
P4	Male	67	4, D	25	4	Yes	Yes	Ex	Retired	Married
P5	Female	68	3, D	18	2	No	No	Ex	Retired	Married
P6	Male	77	2, D	15	2	No	No	Ex	Retired	Married
P7	Male	68	-	-	5	No	No	Ex	Retired	Married
P8	Female	72	2, D	15	2	Yes	Yes	Ex	Retired	Single
P9	Male	74	3, D	25	5	Yes	No	Ex	Retired	Married
P10	Female	74	3, D	29	2	Yes	No	Ex	Retired	Widowed
P11	Female	86	2, D	18	1	No	No	Never	Retired	Widowed
P12	Female	73	3, D	19	1	Yes	No	Ex	Retired	Married
P13	Female	65	1, D	17	1	No	No	Ex	Retired	Single
P14	Male	69	3, D	23	1	No	No	Ex	Retired	Married
P15	Female	63	3, D	10	1	Yes	No	Ex	Retired	Married

*Classification of COPD: Scale 1–4, higher scores indicate greater severity of airflow limitation; Group A to D, D indicates the highest symptom burden and risk of exacerbation (Global Initiative for Chronic Obstructive Lung Disease, 2017).

** CAT: Scale 0–40, higher scores indicate worse patient-reported quality (Global Initiative for Chronic Obstructive Lung Disease, 2017).

*** Charlson index of comorbidity: Scale 0–31, higher scores indicate more comorbidity (Charlson, Pompei, Ales, & MacKenzie, 1987).

**** History of admissions for AECOPD during the study period (excluding the admission when the patient was included).

***** History of readmissions for AECOPD during the study period.

### Data collection

**Figure 1.**
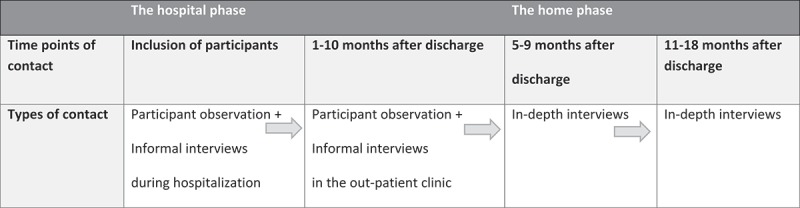
Time points and types of contact for participants.

Data were collected over a period of 18 months, from autumn 2014 to spring 2016. The methods of data collection were participant observations and interviews. The study period and number of participant observations and interviews varied among the participants. The individual study period ranged from four days to 18 months (Figure 1). Out of the 15 patients, 10 completed the entire study. With regard to the five patients who dropped out, three died, one did not have the strength to be interviewed and it was not possible to contact one patient. Because of the design, family members were present during some of the participant observations and interviews. Repeated participant observations and informal interviews were conducted during patient admissions on the respiratory medical ward, at visits to the outpatient clinic and during hospital-based COPD rehabilitation training sessions. Inspired by Spradley (1980), the clinical researcher took an approach that moved from being descriptive to being more focused and selective in observations and questions. During participant observations, fieldnotes (FN) were made in accordance with the nine dimensions proposed by Spradley (1980). The duration of each period of participant observation was between 20 minutes and four hours, according to the situations observed.

In-depth interviews were conducted with 10 patients on two occasions: in the middle and at the end of the study period. Interviews took place in the participants’ own homes, except for two held at the hospital. In conducting serial interviews, the researcher built up a relationship with the participants and focused on changes over time (Murray et al., 2009). The interviews followed a semi-structured interview guide containing questions about how participation in one’s own care arose and developed through the study period. The interview guide was amended for the final interviews to allow relevant topics from the analysis of the earlier interviews to be included. Inspired by recommendations for conducting ethnographic interviews, descriptive questions were combined with structural and contrast questions (Spradley, 1979). The interviews opened with questions like: “How did you experience the time after discharge?”, “How do you manage your own care in daily life?” and “How do you feel supported?” Clarifying questions were added, such as: “What does it mean to you?” The interviews lasted from 14–95 minutes, were digitally recorded and transcribed verbatim.

### Data analysis

Data from FN and interview texts (IW) were transferred to the database programme NVivo version 10 software (QRS International Pty Ltd, Cheshire, UK) to organize the data and facilitate further analysis. A Ricoeur-inspired analysis proceeded on three levels: naïve reading, structural analysis and a critical interpretation and discussion (Dreyer & Pedersen, 2009; Ricoeur, 1976). This process involves using the hermeneutic circle for analysis and interpretation of the text by following its movements from “sense” to “reference,” that is, “from what it says to what it talks about” (Ricoeur, 1976, pp. 87–88), with constant movements between the whole of the text and its parts.

In the naïve reading, texts were read several times in a non-judgmental and open-minded way to achieve an overall expression of the text as a whole. Subsequently, a structural analysis was carried out to confirm and widen the initial understanding (Ricoeur, 1976). The successively gathered data were divided into two parts: the first part consisted of FN and related to what is named “the hospital phase”. The second part was comprised of all the IWs and related to “the home phase” (Saldaña, 2003). Initially, units of meaning with the same content of significance were extracted from the first part of the text and, based on this, themes were formulated. Next, the two parts were compared in a process that centred on how the themes changed over time. The second part was also explored for new themes. In the critical interpretation and discussion, the themes were discussed in relation to nursing theory and other research. Then, the interpretation moved from the specific to the general.

### Ethical considerations

The participants received written and oral information before informed consent was obtained. They were informed that participation was voluntary, that data would be kept confidential and that anonymity was guaranteed (World Medical Association, 2013). During each participant observation and interview, oral information was repeated to ensure that the participants understood that they could withdraw from study at any time without adverse implications for their care. The study was approved by the Danish Data Protection Agency (ID no. 2008–58-0020). In accordance with Danish law, no further ethical approval was required. Throughout the data collection, precautions were taken to protect the participants from distress and physical discomfort. While COPD patients might suffer from breathlessness and fatigue, the length of each series of participant observation and interview was adjusted carefully to their wishes and needs for rest. Furthermore, previous research has shown that repeated data collection can be valuable by giving participants with impaired breathing ability time to tell their stories without stress (Ek, Sahlberg-Blom, Andershed, & Ternestedt, 2011). Thus, in order to reduce the stress for the COPD patients in telling about their experiences, they were met more than once and on several occasions in this study.

## Results

The naïve reading of FN and interview transcripts disclosed values, difficulties, worries and dilemmas, but also opportunities related to the ways that COPD patients approached their illness condition and care, when going through a hospitalization for an AECOPD and returning to their subsequent daily lives at home. At hospital, it seems that the way that patients prepared for the discharge was important to how they managed to take over greater responsibility for their self-management at home. How this unfolded is addressed in the further structural analysis.

The structural analysis is presented in Table 2, wherein the movements between parts and the whole text are illustrated. The two parts show quotations that are representative of the hospital and the home phases, respectively. During the analysis, two main themes and related subthemes emerged. By following the structure of Table 2, themes and subthemes are presented in the following section.

**Table 2.** Examples of the systematic process in the structural analysis.

Units of meaning (Quotations)	Units of significance	Themes and subthemes
**Hospital phase:**		**Struggling to regain a sense of control**
*The patient says to the nurse: “I don’t want to go home yet … I’m too tired …I’ll only fall back into my old ways” (FN, P12)*	Unprepared and seeking to prolong the stay at hospital	Building up strength and readiness for discharge
*“The patient is training on the stairs … he relates that he has*		
*managed to get rehabilitation help, so he can climb the stairs at home … he pumps his fist in his hand” (FN, P6).*	Motivated to influence decisions	
*Patient: “I would like to know if my COPD has got worse.” Doctor: “Yes, that’s the nature of the illness …” The patient later relates that she is happy with the doctor’s explanation (FN, P13)*	Informed appropriately to be able to relate to own situation	Seeking clarity and confidence
*“The nurse is instructing the patient in using a new inhalation medicine … the patient is silent … the nurse encourages her to ask the evening nurse for help … the patient sighs: “If I can just figure it out””(FN, P1)*	Overwhelmed and feeling incompetent	
**Home phase:**		**Striving to comply with received advice and encouragement to maintain well-being**
*Patient: “The doctor said that it was COPD and that I should learn to live with it … I was a bit angry … despondent … now I can’t do this and I can’t do that” (IW P15, 11 months after discharge)*	Discouraged and seeking to renegotiate the meaning of living	Struggling to stay motivated and confident
*Patient: “I had a few sessions with a healthcare person … we made some goals for my daily life … I need structure … and then I can beat the anxiety” (IW P2, 8 months after discharge)*	Empowered to make everyday life work better	
*Patient: “The doctors say that I should go to the doctor in good time, but I try to put it off … it’s stupid, I can see that myself” (IW P9, 6 months after discharge)*	Advised to seek help in good time but reluctant	Asking for help
*The patient relates about her anxiety at night: Patient: “When I was hospitalized, they [the health professionals] suggested a psychologist, but nothing is happening!” Nurse: ”Unfortunately, we don’t have that option. You need to go to your own doctor”* *(FN P5, out-patient clinic, 7 months after discharge)*	Frustrated about not getting the help expected	

### Hospital phase: struggling to regain a sense of control

#### Building up strength

Being hospitalized was something disturbing and uncontrollable, and was expressed as difficult to deal with. During recovery, an increasing desire to go home was shown in the reflections and efforts made to prepare for the discharge. A certain pressure from healthcare professionals putting discharge planning on the agenda meant a risk of feeling forced to relate to one’s situation before feeling ready to leave hospital. Previous demanding recurrences and readmissions were mentioned as incidents that increased the level of worry about discharge. In this case, help from significant others, such as close family members, was of great importance, but this was not without challenges. The following FN points to the worries declared by a patient after a nurse had encouraged her to be discharged the same day. In consideration of her chronically ill husband, whom she was afraid to burden further, the patient begged the nurse to allow her to stay in hospital for one more day: “I don’t want to go home yet … I’m too tired … I’ll only fall back into my old ways” (FN, P12). The statement points to feelings of being unprepared, a fear of recurrence and worries related to a relative’s illness that can lead to attempts to prolong the stay at hospital. Such pressures could visibly increase nervousness and breathlessness. In the case above, the doctor later accepted the request, and the way in which the patient was taken seriously appeared to make her calm. An urge to rest dominated, but a desire to have time enough to increase one’s physical capacities was also present. In such situations, a strong will, but also healthcare professionals’ praise, were mentioned as inciting further activity. The struggle to regain one’s strength was illustrated by this FN: “The patient is training on the stairs … he relates that he has managed to get rehabilitation help, so he can climb the stairs at home … he pumps his fist in his hand” (FN, P6). Both quotations underscore that not only the illness itself, but also conditions related to accommodation and daily living, can motivate patients’ attempts to have an impact on decisions regarding, for example, time of discharge and further rehabilitation. It seems that the strategies used to buy more time, to rest and to train can be interpreted as useful in preparing and dealing with concerns about not being able to manage a worsened health condition and possible recurrences at home. Furthermore, feeling recognized by healthcare professionals for these strategies can be seen as being respected and encouraged to move on.

#### Seeking clarity and confidence

Being capable of understanding what was going on and adapting to new treatments or other recommendations played a central role in preparations for discharge. As patients began to feel better, a growing interest arose in seeking clarity about the current state of their illness, treatment and plans. However, patients listed issues such as breathing problems, lack of energy and unfamiliar language as barriers to engaging in dialogues with healthcare professionals. One patient described how she felt challenged in the communication: “It’s hard when you don’t understand what they are saying, because you only get half of it” (FN, P5). The statement indicates the importance of a common language to feel fully informed and be able to understand and relate to one’s situation. Furthermore, feelings of being overwhelmed and incompetent emerged when instructions on how to handle new inhaler medication and advice about lifestyle changes were incomprehensible. It seems, then, that help to interpret received information is of importance in feeling confident to resume responsibility for one’s own care at home.

In situations involving discord, the challenge could be considerable. In a conversation between a patient and a doctor, one patient wondered why she was not provided with antibiotic treatment for her exacerbation. The doctor answered that, in the current situation, this was not indicated, but that being more physically active and attending a rehabilitation course should be a good solution to her problems. After the conversation, the patient said sadly: “I know it’s important to be active, but it doesn’t help when I get those attacks of breathlessness at home … I feel I’m being put down” (FN, P15). The quotation points to frustrations in not getting the hoped-for help to overcome the well known challenges of daily activity and feelings of being belittled. The quotation also discloses the importance of mutuality in the dialogue between patients and healthcare professionals. To understand not only what they were being told, but also to share and negotiate information and experiences and to feel understood, were seen as essential in relating properly to their own situation and to find healthcare professionals’ suggestions and solutions meaningful.

### Home phase: striving to comply with advice and encouragement to maintain health and well-being

#### Struggling to stay motivated and confident

Similar to the hospital phase, various strategies emerged that patients found useful at home in their efforts to integrate self-management tasks in everyday life. In the struggle to maintain their health and well-being, patients in this study talked about how they strived to pull themselves together to manage their medication, quit smoking, be active and eat as advised. They talked about bad days or periods where they slowed down, dropped their plans for physical and social activities and took one day at a time. On other days, they had more energy and were more active, but then they could find it difficult to economize their energy. The situation became more difficult when concerns about one’s own additional illnesses or relatives’ illnesses dominated and their self-management of COPD was deprioritised. The reactive way in which the daily situation was approached can be interpreted as useful in making day-to-day life manageable, but seems to make it difficult to plan ahead. Although the significance of hospitalization diminished over time, memories of the way in which encounters with healthcare professionals had proceeded could remain. Eleven months after discharge, the patient quoted above related that she still felt uncertain: “The doctor said that it was COPD and that I should learn to live with it … I was a bit angry … despondent … now I can’t do this and I can’t do that” (IW, P15). The quotation points to feelings of discouragement and to difficulties in renegotiating the meaning of living. As here illustrated, feelings of being devalued later gave rise to despondency.

The study indicates that developing a pessimistic attitude to one’s situation could affect the way in which self-management was approached. One patient, who related how she lacked someone to talk to, points to the significance of feeling restricted and isolated. For example, 12 months after discharge and having successfully quit tobacco, she had re-started smoking: “I sit on my own and bore myself to death, so I can just as well smoke myself to death” (IW, P13). The statement points to a dilemma in choosing between a healthy lifestyle and something comforting that the patient associates with well-being. These statements indicate that depressed emotions, lack of acceptance by others, lack of support and a belief that little can be done to improve the situation make it difficult to stay motivated for self-management. In contrast, building up experiences and participation in a local rehabilitation programme had enabled one patient to achieve more balance in her daily life: “I had a few sessions with a healthcare person … we made some goals for my daily life … I need structure … and then I can beat the anxiety” (IW, P2). The statement suggests that being empowered to be more confident in one’s own abilities can be beneficial in dealing with the anxiety associated with the fear of being breathless. Hence, repeated dialogues with healthcare professionals that involve issues related to individuals’ daily lives seem to benefit patients in seeing new possibilities, including learning more pro-active strategies.

#### Asking for help

Knowing when and where to turn for help was shown to be associated with challenges. Although being encouraged by healthcare professionals to seek help in good time, doubts and the desire to manage on one’s own could prevent patients from seeking help. Despite finding it difficult to judge the severity of an exacerbation and facing other health problems, there appeared to be a reluctance to seek help from healthcare professionals. Getting access to the right healthcare professionals could be troublesome and, therefore, adequate and routine follow-up visits were perceived as useful: “They [the health professionals] followed up more this time, so I felt valued. I wasn’t just sent home and forgotten” (IW, P13). The statement indicates that being invited to attend follow ups can raise feelings of being valued and provide patients with what appears to be a lifeline. Willingness to seek help was also seen to be influenced by experiences from previous contact with the healthcare system. The statement: “That’s also why I would prefer not to go to hospital. I don’t want to be a bother” (FN, P10) was a response to a situation in which, after a long waiting time, a patient felt passed over, and points to a desire not to bother the staff. It was obvious that, despite being advised to seek help in good time, some patients preferred to wait and see whether their situation improved without intervention. While such an approach could be understood as appropriate self-management, it could also involve a risk of delaying too long or receiving inadequate help. Further challenges were revealed in the outpatient clinic seven months after discharge, where one patient related that she did not know how to deal with her anxiety at night:

Patient: When I was hospitalized, they [the health professionals] suggested a psychologist, but nothing is happening!

Nurse: Unfortunately, we don’t have that option. You need to go to your own doctor. (FN, P5).

The dialogue allows for an understanding of the frustrations and apathy that can be associated with not getting the help that is required and hoped for. Here, the patient was not aware that she was supposed to take an active role in seeking a psychologist and, as a result, the received suggestion led to nothing. That this was the case was confirmed in a later interview, where the patient still had not managed to seek help. In view of this, it appears that patients can be left to themselves and given a responsibility that they are not able to handle. Thus, their well-being can decrease and instead of being empowered, they seem to be further burdened.

## Discussion

The study shows that the 18-month transitional period from hospitalization to managing day-to-day care at home following a severe AECOPD presents both the opportunity to get a sense of control and challenges around stresses and burdens. The intensity of COPD, the presence of one’s own additional illnesses or relatives’ illnesses and the extent to which the contact with the healthcare system was collaborative are all significant to patients’ perceptions of their capacity to participate actively in their own care—not only in the short-term, but also in the long-term. While collaboration with healthcare professionals in the hospital setting can be seen as providing a helping hand to move on, experiences of lack of collaboration can leave their mark on how well patients self-manage at home. By throwing light on these long-term challenges, this result builds on the existing body of studies about different aspects of COPD patients’ self-management of their condition (Disler et al., 2012).

### The hospital phase

It became obvious that patients in this phase responded to the AECOPD in different ways, depending on their degree of recovery. The struggle to gain a sense of control before discharge manifested itself in both actively and passively-oriented strategies, which patients used to both build up strength and to deal with their concerns about not being ready to leave hospital. A passive strategy before a scheduled discharge was reflected in those patients who preferred to rest and prolong their stay at hospital, while an active strategy was reflected in those patients who took initiative and were engaged in physical activities. These different ways to approach the discharge situation are in line with another study, which showed that, depending on the severity of COPD, patients balanced their daily life with the illness by either battling with it or hiding it (Cooney et al., 2013). The battling methods were associated with pacing strategies to promote activity and with limiting strategies used to avoid breathlessness. In some situations, the latter-mentioned could lead to a restricted life, but in other situations could be useful to conserve energy for the most important actions (Cooney et al., 2013). When preparing for discharge, our study showed that patients used both types of strategy, although limiting strategies dominated the picture. Thus, participating in one’s own care before discharge implies the use of both pacing and limiting strategies to deal with feelings such as doubt and concerns related to taking over the responsibility for one’s own care, while feeling more breathless and weaker than usual.

An important aspect of discharge management and follow-up is that patients are actively involved, ready to look after themselves and, over time, develop their ability to self-manage in ways that involve a positive adaption of their health behaviour and skills to better manage their disease (GOLD, 2017). Our results underscore both the importance and the challenges for patients to reach such levels of competence. In interactional nursing practice theory, nursing is seen as a mutual caring activity, where the nurse and the patient engage in joint activities (Scheel, Pedersen, & Rosenkrands, 2008). The practice theory implies three modes of action. In the cognitive-instrumental approach, problem solving and result-oriented activities related to bodily needs and instrumental activities are in focus, such as complicated inhaler techniques and medication. The aesthetic-expressive approach involves understanding the patient and his/her situation—in our study, related to the situation before and after discharge—and the surrounding environment. The moral-practical mode of action is linked to dialogue and collaboration with the patient in accordance with ethical norms. All three modes of action are integral to the current nursing situation, but the nurse must at any time make a qualified judgement of the current situation to ascertain which mode is the most appropriate (Scheel et al., 2008). Our study shows that the way in which the dialogue with healthcare professionals proceeded was important for patients to feel informed about treatment and plans to an extent that they felt ready to go home. Accordingly, the qualified judgement based on all three modes of action is significant to meet patients’ needs and support their self-management in the discharge management and follow-up process.

We suggested that being recognized by healthcare professionals for the strategies used to prepare for discharge could be encouraging. Getting help to interpret received information, to share and negotiate one’s own understanding of the situation and feeling understood were considered essential for patients to relate properly to their own situation and to find healthcare professionals’ suggestions and solutions meaningful. In relation to interactional nursing practice, any qualified judgement has to be founded on an insight into the patient’s life situation. This implies recognition of the other as a person, and if this is not the case, caring can degenerate into becoming authoritarian, a violation or oppression of the patient (Scheel et al., 2008). Seen in this light, and given that COPD patients admitted with AECOPD are dependent on the judgements of healthcare professionals, it is crucial that the dialogue about readiness for discharge includes patients’ self-assessment of the current situation.

### The home phase

The study pointed to various strategies developed to compensate for limitations and integrate self-management tasks in daily life at home. This is in line with the limiting and pacing strategies identified by Cooney et al. (2013). The use of limiting strategies dominated in our study. This result is in accordance with previous COPD research, which identified the same pattern in the use of behavioural strategies directed at a reduction in activity and a shift from an active to a sedentary lifestyle, which is useful in compensating for breathlessness and fatigue (Cicutto, Brooks, & Henderson, 2004). Such strategies seem to be useful in making everyday life more manageable, but a lack of physical activity over time can also lead to a spiral of physical de-conditioning, fatigue and social isolation (Cicutto et al., 2004; Gullick & Stainton, 2008). Hence, for COPD patients, limiting strategies can be useful and, over time, necessary, but the dominance of such strategies can act as a disincentive to taking an active approach to one’s own care and mean that life becomes more restricted.

Our study showed that the severity of illness does not alone affect the way in which a life with COPD is perceived. Thus, staying motivated for self-management is a challenge faced by almost all patients at some point; however, patients with depressed emotions, lack of acceptance by others and lack of support are particularly at risk of poor self-management motivation. According to Delmar et al. (2006), taking responsibility for oneself implies the freedom to choose. Living with chronic illness implies that there is the opportunity to decide how one shall live with dignity and deal with the conditions at hand. Not choosing is also an option. Making choices is often difficult and can be experienced as a choice between two evils (Delmar et al., 2006). The choice to smoke, as shown in our study, is an example of such a hard choice. Delmar et al. (2006) underscore that self-responsibility not only represents positive values but also involves feelings of self-blame, guilt, punishment and sin. Seen in this light, being responsible and seeking to fit COPD into everyday life can involve difficult decision-making and the emergence of difficult emotions.

The study showed that, besides finding it difficult to stay motivated, patients could also suffer from a lack of confidence in their own ability to integrate self-management into their lives. In our study, this was apparent in the ways in which patients responded to challenges, how they gave up and withdrew from their usual activities and the related feelings of discouragement. Furthermore, when patients did not feel that they were received in a valued and encouraging way, our study identified a long-term uncertainty and difficulties in renegotiating the meaning of living.

From the healthcare professional perspective, a recent study conducted during Danish respiratory outpatient consultations showed that healthcare professionals made a distinction between what they considered to be possible and impossible topics for counselling. For example, when they strived to focus on the diseased lungs, this created further suffering in patients but also a discomfort and frustration among healthcare professionals (Jensen, Larsen, & Konradsen, 2016). When understood in the light of interactional nursing practice, such challenges highlight the importance of the aesthetic-expressive mode of action, because it aims for a mutual understanding in the dialogue, on which all parties can reflect, leading to good solutions that are incorporated into the patient’s life situation. In our study, patients furthermore expressed that they felt valued when they were invited to follow-up visits after a hospitalization, rather than having to take the initiative. Hence, being invited to have iterative dialogues with healthcare professionals can be essential for patients to learn about new possibilities, including more pro-active strategies.

The patients in our study encountered some difficulty in dealing with their need for help. Although they were encouraged to seek help in good time, doubts and a desire to manage on their own could dominate their decision making. In one way, such an attitude can be seen as appropriate self-management behaviour, but it could also involve the risk of delayed or inadequate help. A Dutch study indicated that patients with COPD to a minor-to-severe degree were challenged in recognition of exacerbations and, consequently, in taking adequate self-management action, such as seeking help. Having trust in healthcare professionals was one of the factors that influenced how patients approached these challenges (Korpershoek, Vervoort, Nijssen, Trappenburg, & Schuurmans, 2016). Other research on patients’ needs following a hospital discharge after an AECOPD points to a need for personalized self-management advice about when and how to call for help (Gruffydd-Jones, Langley-Johnson, Dyer, Badlan, & Ward, 2007). Delmar et al. (2006) showed that seeking help implies a balance between being dependent on help from others and independence of help from others. Thus, seeking help can imply a difficult choice between maintaining control and handing control over to others.

Our findings reveal that patients showed an interest in participating actively in their care, including collaborating with healthcare professionals, both during hospitalization and at home, but that they needed to be adequately supported. Scheel et al. (2008) argue that self-care and care cannot be seen independently of one another. From their perspective, self-care is essential to strengthening the patient’s ability to exercise self-help, but they warn against making self-care into a superordinate and governing concept and translating it into practice without care. In practice, nurses must be able both to practise care and to support self-care (Scheel et al., 2008). Judgements must be made in each situation, to find out when to practise either care or self-care, or both. When patients in our study were unable to follow up as expected by healthcare professionals, it meant that they were left to themselves and to a responsibility, that they were unable to assume. Thus, encouraging patients to participate actively in their own care requires easily accessible and iterative interactions between patients and healthcare professionals, who are sensitive to the patients’ own view on their situation, but also to ethical dimensions concerning whether patients are capable of taking or willing to take responsibility for their own health.

### Study limitations and strengths

One limitation might be that the study was based in a single hospital, because discharge management and follow-up strategies differ between institutions. The small number of participants in the study might constitute another limitation. However, conducting repeated data collection in different care and home contexts over 18 months was of great value to achieve the breadth of the experience of being a participant in one’s own care (Polit & Beck, 2006). According to Murray et al. (2009), there is a risk in longitudinal qualitative research to generate a large volume of data and thus, making the analysis process unmanageable. Hence, the initial number of 15 patients was considered sufficient to maintain the overview and depth of the data analysis.

Both the prolonged data collection and the use of participant observations and interviews as complementary methods has resulted in rich data, which is supposed to have deepened the analysis and our understanding. According to Ricoeur (1976), it is always possible to argue for and against an interpretation. The results should therefore be considered to represent one possible way of understanding experiences related to COPD patients’ participation in their own care. Through the analysis process, we have tried to be open-minded and reflect critically on each other’s interpretations. To this end, reflections were made to become more aware of our preconceptions. Furthermore, discussing the results of the analysis several times with colleagues in the field has contributed to strengthening the study.

## Conclusion

The study shows that, from the COPD patient’s point of view, participating in one’s own care during the transition from hospitalization to daily life at home following an AECOPD is linked to being properly involved in decisions about one’s care and being able to perform self-management in line with healthcare professionals’ recommendations. Staying motivated for self-management is a complex process that involves patient-related factors, healthcare-professional-related factors and the interaction between patients and healthcare professionals. When not properly supported, the requirement to take over a greater level of responsibility for one’s own care can place an additional burden on patients. With more knowledge of the significance of patients’ participation in their own care, healthcare professionals can be more sensitive to patients’ specific care and support needs and thereby contribute to promoting their health and well-being.

## Clinical implications

The study emphasizes that knowing and valuing COPD patients’ perspective and knowledge is essential for clinical judgement and effective therapeutic caring. Involving patients in training about how to manage early exacerbation symptoms appears to be an essential part of discharge management. In this case, personalized care planning, in which patients are encouraged to participate in goal setting and action planning, can be an appropriate tool to support better self-management (Trappenburg et al., 2011). Developing and implementing easily accessible and flexible follow-up pathways might support patients in self-managing their condition over time. Integrated interventions by telemedicine, such as telemedicine video consultations with nurses placed at hospital and patients at home, could be a possible solution that, furthermore, might contribute to overall efforts to reduce readmissions (Sorknaes, Madsen, Hallas, Jest, & Hansen-Nord, 2011).

## References

[CIT0001] Abad-Corpa E., Royo-Morales T., Iniesta-Sánchez J., Carrillo-Alcaraz A., Rodríguez-Mondejar J. J., Saez-Soto A. R., & Vivo-Molina M. C. (2013). Evaluation of the effectiveness of hospital discharge planning and follow-up in the primary care of patients with chronic obstructive pulmonary disease. *Journal of Clinical Nursing*, 22(5–6), 669–13. doi:10.1111/j.1365-2702.2012.04155.x 22830974

[CIT0002] Angel S., & Frederiksen K. N. (2015). Challenges in achieving patient participation: A review of how patient participation is addressed in empirical studies. *International Journal of Nursing Studies*, 52(9), 1525–1538. doi:10.1016/j.ijnurstu.2015.04.008 25980558

[CIT0003] Bailey C., Hewison A., Karasouli E., Staniszewska S., & Munday D. (2016). Hospital care following emergency admission: A critical incident case study of the experiences of patients with advanced lung cancer and Chronic Obstructive Pulmonary Disease. *Journal of Clinical Nursing*, 25(15–16), 2168–2179. doi:10.1111/jocn.13170 27139373

[CIT0004] Bourbeau J. (2008). Clinical decision processes and patient engagement in self-management. *Disease Management & Health Outcomes*, 16(5), 327–333. doi:10.2165/0115677-200816050-00009

[CIT0005] Bourbeau J., Saad N., Joubert A., Ouellet I., Drouin I., Lombardo C., … Lebel M. (2013). Making collaborative self-management successful in COPD patients with high disease burden. *Respiratory Medicine*, 107(7), 1061–1065. doi:10.1016/j.rmed.2013.03.003 23541484

[CIT0006] Castro E. M., Van Regenmortel T., Vanhaecht K., Sermeus W., & Van Hecke A. (2016). Patient empowerment, patient participation and patient-centeredness in hospital care: A concept analysis based on a literature review. *Patient Education & Counseling*, 99(12), 1923–1939. doi:10.1016/j.pec.2016.07.026 27450481

[CIT0007] Charlson M. E., Pompei P., Ales K.-L., & MacKenzie C.-R. (1987). A new method of classifying prognostic comorbidity in longitudinal studies: Development and validation. *Journal of Chronic Diseases*, 40(5), 373–383. doi:10.1016/0021-9681(87)90171-8 3558716

[CIT0008] Chen K., Chen M., Lee S., Cho H., & Weng L. (2008). Self-management behaviours for patients with chronic obstructive pulmonary disease: A qualitative study. *Journal of Advanced Nursing*, 64(6), 595–604. doi:10.1111/j.1365-2648.2008.04821.x 19120574

[CIT0009] Chen K.-H., Liu C.-Y., ShyuYea-Ing L., & Yeh S.-L. (2016). Living with chronic obstructive pulmonary disease: The process of self-managing chronic obstructive pulmonary disease. *Journal of Nursing Research (Lippincott Williams & Wilkins)*, 24(3), 262–271. doi:10.1097/jnr.0000000000000152 27015593

[CIT0010] Cicutto L., Brooks D., & Henderson K. (2004). Self-care issues from the perspective of individuals with chronic obstructive pulmonary disease. *Patient Education & Counseling*, 55(2), 168–176. doi:10.1016/j.pec.2003.08.012 15530751

[CIT0011] Cooney A., Mee L., Casey D., Murphy K., Kirwan C., Burke E., … Murphy J. (2013). Life with chronic obstructive pulmonary disease: Striving for ‘controlled co-existence’. *Journal of Clinical Nursing*, 22(7–8), 986–995. doi:10.1111/j.1365-2702.2012.04285.x 23279604

[CIT0012] Coulter A. (2011). *Engaging patients in healthcare*. Milton Keynes: Open University Press.

[CIT0013] Coulter A., Entwistle V. A., Eccles A., Ryan S., Shepperd S., & Perera R. (2015). Personalised care planning for adults with chronic or long-term health conditions. *Cochrane Database of Systematic Reviews*, 3, N.PAG-N.PAG. doi:10.1002/14651858.CD010523.pub2 PMC6486144 25733495

[CIT0014] Danish Health Authority, Sundhedsstyrelsen (2015). *Anbefalinger for tværsektorielle forløb for mennesker med KOL*. Retrieved 4 2, 2017, from http:///AppData/Local/Microsoft/Windows/INetCache/IE/33EQ21O8/KOLanbefForlÃ¸b_18dec15ufigurer.pdf

[CIT0015] Delmar C., Bøje T., Dylmer D., Forup L., Jakobsen C., Møller M., … Pedersen B. D. (2006). Independence/dependence – A contradictory relationship? Life with a chronic illness. *Scandinavian Journal of Caring Sciences*, 20(3), 261–268. doi:10.1111/j.1471-6712.2006.00403.x 16922979

[CIT0016] Disler R., Appleton J., Smith T. A., Hodson M., Inglis S. C., Donesky D., & Davidson P. M. (2016). Empowerment in people with COPD. *Patient Intelligence*, 8(7–20). doi:10.2147/PI.S61195

[CIT0017] Disler R. T., Gallagher R. D., & Davidson P. M. (2012). Factors influencing self-management in chronic obstructive pulmonary disease: An integrative review. *International Journal of Nursing Studies*, 49(2), 230–242. doi:10.1016/j.ijnurstu.2011.11.005 22154095

[CIT0018] Dreyer P. S., & Pedersen B. D. (2009). Distanciation in Ricoeur’s theory of interpretation: Narrations in a study of life experiences of living with chronic illness and home mechanical ventilation: Feature. *Nursing Inquiry*, 16(1), 64–73. doi:10.1111/j.1440-1800.2009.00433.x 19228305

[CIT0019] Ek K., Sahlberg-Blom E., Andershed B., & Ternestedt B.-M. (2011). Struggling to retain living space: Patients’ stories about living with advanced chronic obstructive pulmonary disease. *Journal of Advanced Nursing*, 67(7), 1480–1490. doi:10.1111/j.1365-2648.2010.05604.x 21375574

[CIT0020] Giacomini M., DeJean D., Simeonov D., & Smith A. (2012). Experiences of living and dying with COPD: A systematic review and synthesis of the qualitative empirical literature. *Ontario Health Technology Assessment Series*, 12(13), 1–47. PMC3384365 23074423

[CIT0021] Global Initiative for Chronic Obstructive Lung Disease (GOLD) (2017). *Global strategy for the diagnosis, management and prevention of COPD*. Retrieved from http://goldcopd.org

[CIT0022] Gruffydd-Jones K., Langley-Johnson C., Dyer C., Badlan K., & Ward S. (2007). What are the needs of patients following discharge from hospital after an acute exacerbation of chronic obstructive pulmonary disease (COPD)? *Primary Care Respiratory Journal : Journal of the General Practice Airways Group*, 16(6), 363–368. doi:10.3132/pcrj.2007.00075 PMC6634232 18038104

[CIT0023] Gullick J., & Stainton M. C. (2008). Living with chronic obstructive pulmonary disease: Developing conscious body management in a shrinking life-world. *Journal of Advanced Nursing*, 64(6), 605–614. doi:10.1111/j.1365-2648.2008.04823 19120575

[CIT0024] Harrison S. L., Janaudis-Ferreira T., Brooks D., Desveaux L., & Goldstein R. S. (2015). Self-management following an acute exacerbation of COPD: A systematic review. *CHEST*, 147(3), 646–661. doi:10.1378/chest.14-1658 25340578

[CIT0025] Hounsgaard L., Petersen L. K., & Pedersen B. D. (2007). Facing possible illness detected through screening–experiences of healthy women with pathological cervical smears. *European Journal of Oncology Nursing*, 11(5), 417–423. doi:10.1016/j.ejon.2007.04.005 17604694

[CIT0026] Jensen A. L., Vedelø T. W., & Lomborg K. (2013). A patient-centred approach to assisted personal body care for patients hospitalised with chronic obstructive pulmonary disease. *Journal of Clinical Nursing*, 22(7–8), 1005–1015. doi:10.1111/jocn.12050 23331341

[CIT0027] Jensen L., Brinkjaer U., Larsen K., & Konradsen H. (2015). Exploring the unmet needs of the patients in the outpatient respiratory medical clinic: Patients versus clinicians perspectives. *International Journal of Chronic Diseases Print*, 2015, 749369. 10.1155/2015/749369 PMC4689906 26783555

[CIT0028] Jensen L., Larsen K., & Konradsen H. (2016). Maintaining a distinction between possible and impossible topics of conversation in the outpatient respiratory medical clinic. *Global Qualitative Nursing Research*, 3(1–12). doi:10.1177/2333393616638977 PMC5342844 28462333

[CIT0029] Jolly K., Majothi S., Sitch A. J., Heneghan N. R., Riley R. D., Moore D. J., … Jordan R. E. (2016). Self-management of health care behaviors for COPD: A systematic review and meta-analysis. *International Journal of COPD*, 11, 305–326. doi:10.2147/COPD.S90812 26937183 PMC4762587

[CIT0030] Jonkman N. H., Westland H., Trappenburg J. C. A., Groenwold R. H. H., Bischoff E. W. M. A., Bourbeau J., … Schuurmans M. J. (2016). Do self-management interventions in COPD patients work and which patients benefit most? An individual patient data meta-analysis. *International Journal of COPD*, 11(1), 2063–2074. doi:10.2147/COPD.S107884 27621612 PMC5012618

[CIT0031] Jonsdottir H. (2013). Self-management programmes for people living with chronic obstructive pulmonary disease: A call for a reconceptualisation. *Journal of Clinical Nursing*, 22(5–6), 621–637. doi:10.1111/jocn.12100 23398312

[CIT0032] Korpershoek Y. J. G., Bos-Touwen I. D., De Man-Van Ginkel J. M., Lammers J. W. J., Schuurmans M. J., & Trappenburg J. C. A. (2016). Determinants of activation for self-management in patients with COPD. *International Journal of COPD*, 11(1), 1757–1766. doi:10.2147/COPD.S109016 27536087 PMC4976914

[CIT0033] Korpershoek Y. J. G., Vervoort S. C. J. M., Nijssen L. I. T., Trappenburg J. C. A., & Schuurmans M. J. (2016). Factors influencing exacerbation-related self-management in patients with COPD: A qualitative study. *International Journal of COPD*, 11(1), 2977–2990. doi:10.2147/COPD.S116196 27932877 PMC5135062

[CIT0034] Kvangarsnes M., Torheim H., Hole T., & Ohlund L. S. (2013a). Intensive care unit nurses’ perceptions of patient participation in the acute phase of chronic obstructive pulmonary disease exacerbation: An interview study. *Journal of Advanced Nursing*, 69(2), 425–434. doi:10.1111/j.1365-2648.2012.06021.x 22512673 PMC3594967

[CIT0035] Kvangarsnes M., Torheim H., Hole T., & Ohlund L. S. (2013b). Narratives of breathlessness in chronic obstructive pulmonary disease. *Journal of Clinical Nursing*, 22(21–22), 3062–3070. doi:10.1111/jocn.12033 23889291 PMC4229005

[CIT0036] Lange P. (2012). Chronic care for COPD patients in Denmark. *Pneumonologia i Alergologia Polska*, 80(4), 292–295. 22714071

[CIT0037] Lomborg K., & Kirkevold M. (2008). Achieving therapeutic clarity in assisted personal body care: Professional challenges in interactions with severely ill COPD patients. *Journal of Clinical Nursing*, 17(16), 2155–2163. doi:10.1111/j.1365-2702.2006.01710.x 18710375

[CIT0038] Maslowski J. (2012). Patient participation: An emerging nursing issue. *International Journal for Human Caring*, 16(4), 74–78.

[CIT0039] Murray S. A., Kendall M., Carduff E., Worth A., Harris F. M., Lloyd A., … Sheikh A. (2009). Use of serial qualitative interviews to understand patients’ evolving experiences and needs. *BMJ*, 339, b3702. doi:10.1136/bmj.b3702 19786485

[CIT0040] Polit D. F., & Beck C. T. (2006). *Essentials of nursing research: Methods, appraisal, and utilization* (6th ed.). Philadelphia, PA: Lippincott Williams & Wilkins.

[CIT0041] Ricoeur P. (1976). *Interpretation theory: Discourse and the surplus of meaning* (6th ed.). Fort Worth, TX: Texas Christian University Press.

[CIT0042] Saldaña J. (2003). *Longitudinal qualitative research: Analyzing change through time*. Walnut Creek, CA: AltaMira Press.

[CIT0043] Scheel M. E., Pedersen B. D., & Rosenkrands V. (2008). Interactional nursing - A practice-theory in the dynamic field between the natural, human and social sciences. *Scandinavian Journal of Caring Sciences*, 22(4), 629–636. doi:10.1111/j.1471-6712.2007.00564.x 19068053

[CIT0044] Sørensen D., Frederiksen K., Grøfte T., & Lomborg K. (2013). Practical wisdom: A qualitative study of the care and management of non-invasive ventilation patients by experienced intensive care nurses. *Intensive & Critical Care Nursing*, 29(3), 174–181. doi:10.1016/j.iccn.2012.10.001 23159242

[CIT0045] Sorknaes A. D., Madsen H., Hallas J., Jest P., & Hansen-Nord M. (2011). Nurse tele-consultations with discharged COPD patients reduce early readmissions - an interventional study. *Clinical Respiratory Journal*, 5(1), 26–34. doi:10.1111/j.1752-699X.2010.00187.x 21159138

[CIT0046] Spradley J. P. (1979). *The ethnographic interview*. Belmont, CA: Wadsworth Group.

[CIT0047] Spradley J. P. (1980). *Participant observation*. Fort Worth: Harcourt Brace College Publishers.

[CIT0048] Steer J., Gibson G. J., & Bourke S. C. (2010). Predicting outcomes following hospitalization for acute exacerbations of COPD. *QJM*, 103(11), 817–829. doi:10.1093/qjmed/hcq126 20660633

[CIT0049] Steiner M. (2015). Hospital admission and readmission for acute exacerbation of COPD. A tough nut to crack. *Thorax*, 70(12), 1108–1109. doi:10.1136/thoraxjnl-2015-207986 26567184

[CIT0050] Tobiano G., Marshall A., Bucknall T., & Chaboyer W. (2015). Patient participation in nursing care on medical wards: An integrative review. *International Journal of Nursing Studies*, 52(6), 1107–1120. doi:10.1016/j.ijnurstu.2015.02.010 25769475

[CIT0051] Torheim H., & Kvangarsnes M. (2014). How do patients with exacerbated chronic obstructive pulmonary disease experience care in the intensive care unit? *Scandinavian Journal of Caring Sciences*, 28(4), 741–748. doi:10.1111/scs.12106 24313779 PMC4260168

[CIT0052] Trappenburg J. C. A., Monninkhof E. M., Bourbeau J., Troosters T., Schrijvers A. J. P., Verheij T. J. M., & Lammers J. W. J. (2011). Effect of an action plan with ongoing support by a case manager on exacerbation-related outcome in patients with COPD: A multicentre randomised controlled trial. *Thorax*, 66(11), 977–984. doi:10.1136/thoraxjnl-2011-200071 21785156

[CIT0053] Wagner E.H. (1998). Chronic disease management: what will it take to improve care for chronic illness? effective clinical practice. 1(1), 2–4. 10345255

[CIT0054] World Health Organization (WHO) (2017). Chronic respiratory diseases. Chronic obstructive pulmonary disease: Retrieved from http://www.who.int/respiratory/copd/en/

[CIT0055] World Medical Association (2013). World medical association declaration of helsinki: Ethical principles for medical research involving human subjects. *JAMA*, 310(20), 2191–2194. doi:10.1001/jama.2013.281053 24141714

[CIT0056] Zwerink M., Brusse-Keizer M., van der Valk P. D., Zielhuis G. A., Monninkhof E. M., van der Palen J., … Effing T. (2014). Self management for patients with chronic obstructive pulmonary disease. *The Cochrane Database of Systematic Reviews*, 3, CD002990. 10.1002/14651858.CD002990.pub3 PMC7004246 24665053

